# Cumulative health disadvantages: an empirical study of the health and mobility of the first cohort of migrant workers in China

**DOI:** 10.3389/fpubh.2023.1221082

**Published:** 2023-07-31

**Authors:** Fengxian Qiu, Qingyang Kong, Dongjun Fan

**Affiliations:** ^1^Department of Social Work, School of Law, Anhui Normal University, Wuhu, China; ^2^School of Economics and Management, Anhui Normal University, Wuhu, China

**Keywords:** the first cohort of migrant workers, cumulative disadvantage, life course, mobility, health

## Abstract

**Background:**

The study aimed to understand the factors affecting the health and healthcare of the first cohort of migrant workers in China using the concept of the cumulative disadvantage framework.

**Methods:**

Data from the China Migrants Dynamic Survey (2017) were used to analyze the process of cumulative disadvantage of health and healthcare among migrant workers. The study also analyzed the spatial lag problem between localized medical insurance policies and healthcare accessibility.

**Results:**

The results revealed a significant negative association between the mobility of the first cohort of migrant workers and their health status. Long-term exposure to hazardous work had a greater negative impact on their health. Chinese migrant workers faced significant obstacles in accessing healthcare due to the lack of portability in health insurance.

**Conclusion:**

The study emphasizes the urgent need for addressing the structural barriers hindering healthcare access and outcomes for migrant workers. It is crucial to promote a more equitable and sustainable healthcare system in China to ensure migrant workers’ health and well-being.

## 1. Introduction

Migrant workers have made significant contributions to China’s economic and social development, improving their families’ overall living conditions and alleviating rural poverty, while effectively restraining the rapid development of urban/rural disparities in the economy (1). However, as a socially vulnerable group (2), migrant workers face difficulties arising from lagging social policies and a lack of social rights (3), particularly in terms of health issues (4). As of the end of 2021, there were approximately 292 million migrant workers in China, accounting for 39% of China’s total employed population (5). The average age of migrant workers is 41.7 years old, with those aged 40 and below accounting for 48.2%, and those aged 40–50 accounting for 27.3% of all migrant workers (6). The concept of the first cohort of migrant workers refers to the rural population who were born before 1980 and started to work outside around 1985 (7). In the 1980s, the number of floating population in China rapidly increased as a result of accelerated industrialization and urbanization processes, leading to the largest-scale influx of rural migrant workers in Chinese history, often referred to as the “migrant worker wave.” (8) These mobile populations were the first group of rural farmers to enter cities in search of employment opportunities following China’s reform and opening-up policy. They migrated to urban areas with the aim of improving their family’s living conditions and made significant contributions to China’s economic development (1). However, due to household registration restrictions, they rarely obtained urban residency status or enjoyed the social welfare benefits provided by the urban social welfare system (3). They remained registered as rural residents, solely working in urban areas, and would return home when there were no job opportunities available (4). While working in cities, they saved as much as possible, minimizing their own expenses, and accumulated income to support their children’s education, schooling, and marriage. They primarily focused on their family’s needs and neglected their own well-being. This distinctive characteristic of the first-cohort migrant workers in China represents their commitment to sacrificing personal expenses for the sake of their family’s welfare (9).

On the other hand, the second cohort of migrant workers refers to those born after 1980 and who entered urban areas for work after 2,000 year. Their motivation for migrating to cities is more driven by personal development and the pursuit of an urban lifestyle, with economic gains no longer being their primary objective (10).

Thus, the trend of migrant workers toward aging is apparent, and as a 4D (dirty, dangerous, damaging, and difficult) employment group (11), they face greater health risks and challenges than the general population and have a lower capacity to cope with health risks (12).

This study was based on the unique life experiences of the first cohort of migrant workers. Using the data of CMDS (2017), with migrant workers’ self-rated health as the dependent variable and spatial and temporal dimensions as analytical perspectives, this study adopted a life course approach to construct a theoretical framework for analyzing the first cohort of migrant workers’ health.

Can existing health care policies protect their health? What factors influence their health? This paper uses quantitative method to examine factors influencing the first cohort of migrant workers health, specifically, we inquire what will they rely on? In the process, we shed light on the social rights, social stratification systems, and cumulative disadvantage in health as well as the access and management of healthcare in China.

### 1.1. The accumulation of disadvantages and life course

Flaskerud and Winslow defined the concept of vulnerable groups as groups that lack sufficient resources and have higher rates of illness and death. They mainly considered the health status of a particular group in a specific social structure, emphasizing the influence of society on individual health maintenance and social rights (13). In the definition of the concept, the core indicators of vulnerable groups are their ability to access resources, their health status, and the risks they face. This concept has been applied to research on the health statuses of various groups, and discussed regarding the relationship between health status and its effects. One theory of cumulative disadvantage is closely related to the concept of vulnerable groups; this theory posits that vulnerable groups acquire typical cumulative disadvantages throughout their life course (14). The focus of the theory of cumulative disadvantage is to identify the process by which opportunities for inequality lead to the continuous accumulation of a disadvantaged status throughout an individual’s life (15). O’Rand believes that a person’s social status and related benefits widen the differences between individuals or groups over time and with age, leading to the accumulation of disadvantages. Currently, the theory of cumulative disadvantage is often used in conjunction with life course theory (16). Many researchers have focused on the accumulation of disadvantages when exploring individuals’ life courses (17). In essence, an important potential theme in life course analysis is the concept of accumulating advantages (or disadvantages), which means that individuals obtain privileges (or disadvantages) based on their social status in early life (18). People in different groups occupy different positions in occupational development and hierarchical structures, so their vulnerability to social order or livelihood opportunities resulting from large-scale social changes is also different (15). Social system changes have different effects on individuals in different life stages, and thus, different age groups are significantly affected by the unique impact of this social change process, which is, at least in part, determined by their advantageous (or disadvantageous) status in the social structure (19). Therefore, the life course can be used as a unique age-stratified indicator of risks and a sign of a unique and cohort-related social position in the opportunity structure, forming a “risk chain” (20). At the same time, childhood experiences affect individuals’ adult physical and mental health (21, 22). In adulthood, there is a high correlation between social and economic status, lifestyle, and health status (23, 24). The life course is a carrier that indicates different roles, responsibilities, and expectations at different ages (25, 26). An individual’s life course is shaped by the interaction between social mechanisms and individual traits, among which “turning point events” (27), such as migration and retirement, are the markers that can change the direction of an individual’s life trajectory (28). Therefore, poverty in older individuals is the result of the accumulation of disadvantages throughout their life course (29, 30). This perspective links individuals’ temporary states at different stages of life and different observation points to form a continuous life course (31). In recent years, the life course perspective has shown significant advantages in explaining the poverty status of older people (32, 33), indicating that older people’s poverty can be attributed not only to current conditions, but also to the accumulation of disadvantages throughout their lives, especially in those with sustained negative life experiences (34). This perspective attempts to bridge a gap between individuals at the micro level and at the macro level, integrating accumulated and constructed life courses.

### 1.2. Migrant workers’ economic income and health maintenance ability

Research on the relationship between health and income has primarily focused on industrialized countries, with most findings strongly supporting the impact of health on employment or income (35, 36). From a micro perspective, a possible mechanism by which health affects income is that healthy individuals can directly increase labor productivity (37), whereas changes in work habits during illness may lead to a suboptimal income function and result in income loss (38). There is a positive correlation between health and economic income, wherein a good health status promotes income, and a significant deterioration in health reduces household income (39). Income, in turn, affects health development through living standards and medical conditions (40), thus forming a cyclical system between the health and income of migrant workers (41, 42). Compared with those in good health, migrant workers with poor health earn only 63.43% of their average annual income from migrant work (43). In terms of the use of medical resources by migrant workers (44), research has found that the probability of low-income groups using the healthcare system is much lower than that of high-income groups (45, 46). Feng and Yu found that the effect of income inequality on health has a lagging effect and can be described as a “U-shaped” relationship (47), where the impact of income inequality on health is mainly negative when income inequality is high (48).

### 1.3. The accumulation of healthy weaknesses in of migrant workers

The Grossman health capital theory suggests that health is not only an investment; it is also a consumption good. While each person’s initial endowment of health may differ, their personal choices can affect their subsequent level of health, and their socio-economic status and educational level can also cause differences in health production (49).

Due to their lack of innate education, rural migrant workers who enter the secondary labor market face obvious environmental injustice issues, as their living and working conditions are often affected by severe pollution (50). The health risks associated with the hazardous environment to which they are exposed include infectious diseases, production accidents, and occupational hazards, all of which threaten the health and life of rural migrant workers (51). Furthermore, long working hours and engagement in high-intensity physical labor negatively impact their health (52, 53). Research shows that the overall health levels of rural migrant workers declined during the period of 1997–2006. As a result of this health decline, rural migrant workers often chose to return to their hometowns for economic reasons (54). However, compared to rural medical conditions, urban medical conditions have a greater effect on promoting the health of rural migrant workers. Therefore, this choice further exacerbates the health vulnerability of rural migrant workers (55).

### 1.4. Medical insurance and utilization of medical services for migrant workers

The medical insurance system in China shows a relationship with social identity, with regional and identity differences being noted, which leads to differences in the effectiveness of medical services among different populations and regions (56). Research by Ye Minghua using macro-level data from China showed that, from 1991 to 2000, medical services in both urban and rural areas were considered luxury goods. It was not until the establishment of the new rural cooperative medical care system in rural areas that medical services became a necessity for rural residents in 2004, allowing them to enjoy medical service benefits with very low incomes (57). The new rural cooperative medical care system has increased farmers’ utilization of medical services and significantly improved the health of high-income populations, while the impact on middle- and low-income participating farmers has not been significant (58). In a study of healthcare resource utilization among rural migrant workers, Wang et al. found that 11% of migrant workers had never sought medical services when they fell ill, 65% chose self-medication, and although 24% chose to seek medical attention, 48% of them went to unlicensed private clinics or grassroots health organizations. Only when faced with serious illnesses were these rural migrant workers forced to seek medical services. Among those who needed hospitalization, 30% refused treatment, and 23% chose to return to their hometown or elsewhere for medical treatment (59). In academic research, scholars have paid more attention to and primarily discussed the relationship between migrant workers’ insurance participation and health. There has been less discussion about the spatial separation and health relationships associated with the first cohort of rural migrant workers’ health maintenance.

### 1.5. Movement and health of migrant workers

In studies on immigrant health and duration of stay, there is a phenomenon known as the “salmon bias effect” or “healthy migrant effect,” which refers to the inverse health selection effect that exists among immigrants during the process of migration (60). This effect suggests that the health status of migrants is selectively better than that of other residents in their place of origin and the general population (61), due to the selective migration of healthier individuals during the migration process However, due to the influence of factors such as lifestyle, health behaviors, and socioeconomic status at the destination, this health advantage gradually diminishes over time (62). Therefore, immigrants whose health conditions significantly deteriorate are often unable to stay in their destination countries for a long time. Some studies have also pointed out that the link between migration and health is mainly due to the pressure associated with migration.

In the research on the migration behavior and health of Chinese rural migrant workers, Liu Juanjuan studied the impact of health risks on rural labor migration and found that, the greater the health risk, the less willing rural laborers are to migrate outwards. Those who perceive themselves to be in better health are more likely to seek work opportunities outside their hometowns (63).

According to survey data on rural migrant workers in the eight districts of Beijing collected by Yuan Huina, approximately one-quarter of the migrant workers experienced a decline in their health status after migration. The study also found a cyclical relationship between health and income among migrant workers (39). Specifically, those who initially had better health statuses had a higher unit earnings rate, but declines in their health statuses led to a decrease in their unit earnings rate (37). Moreover, rural migrant workers with lower socioeconomic statuses were more likely to experience a declines in health (42).

A similar cyclical effect was also found in a study by Yin Qing (64) on the relationship between health and income among rural residents in China. The present study discusses the relationship between the mobility of migrant workers in China and their health. It is noted that migrant workers in China have high mobility, in terms of both geographic and occupational mobility. While this mobility can be seen as upward and self-selected, it also comes with instability, risk, and stress, which can affect the health of migrants. Measures of cross-provincial migration and job changes are used to reflect migrant workers’ mobility, as well as the length of time spent away from their hometowns.

It should be noted that research has shown that the deterioration of migrant workers’ health can place a heavy burden on both the individual and their families. In addition, we suggest that the burden of ill health can be transferred to rural areas when sick migrant workers return home, exacerbating the urban/rural health gap. Existing research has focused on various factors related to migrant workers’ health, such as healthcare systems, social capital, income, occupational diseases, and work-related injuries. However, there is a lack of theoretical frameworks that comprehensively examine migrant workers’ health issues.

The current study suggests that analyzing the health of migrant workers from a generational perspective may be useful, as there are differences between the health of the first cohort of migrant workers and that of second-generation migrant workers. The unique life experiences of the first cohort of migrant workers and their environment mean that their health statuses and the factors that affect it are different from those of second-generation migrant workers.

This research aimed to fill gaps in the existing literature by focusing on the health of the first cohort of migrant workers and introducing the concept of “health vulnerability accumulation.” We aimed analyze the health status of the first cohort of migrant workers in detail, focusing on several dimensions, such as the accumulation of health vulnerability over time, and spatial lags in healthcare insurance, occupational differences, and work duration.

Based on the above analysis, this study proposes the following hypotheses:

*Hypothesis 1:* The spatial lag effect of medical insurance affects the ability of migrant workers to maintain their health.

*Hypothesis 2:* The cumulative disadvantages of the first cohort of migrant workers in terms of health are related to their length of employment.

*Hypothesis 3:* Sacrificing health for income is an unavoidable choice for migrant workers.

## 2. Study design and data description

### 2.1. Construction of the model of cumulative disadvantages for the first cohort of migrant workers

This study, which is based on an individual life course perspective, discusses the influence of social factors on personal life opportunities. Given the unique life course of the first cohort of migrant workers, especially their disadvantaged position as social outsiders and weak actors in the labor market, they have become a vulnerable group in society due to the accumulation of factors such as age, skills, health, and social exclusion, exhibiting typical characteristics of the accumulation of disadvantages in health. We attempted to analyze the uniqueness and related factors of the health problems of the first cohort of migrant workers using a disadvantaged accumulation model that is highly compatible with their life course, and by exploring the factors that affect their health maintenance during the process of migration for work.

Due to the lack of long-term tracking survey data, this study selected cross-sectional data to establish a targeted explanatory framework for the factors that influence the health of the first cohort of migrant workers based on relevant indicators and data. Although the first cohort of migrant workers is highly suited to the conceptual model of vulnerable groups in terms of livelihood, resource accessibility, health status, and risk risk-coping ability, due to limitations in length and research topics, this study mainly discussed the process of health disadvantage accumulation among the first cohort of migrant workers, temporarily leaving the issues of livelihood resource accessibility and risk-coping ability for further specialized research.

Disadvantage accumulation is the process by which the disadvantaged status that results from unequal opportunities accumulates over the course of a person’s life (65). As a person’s social status and related interests change over time and with age, differences between individuals or groups become more pronounced (13). The key factor in the accumulation of disadvantages in the migrant worker group in this study is the time-based disadvantage accumulation of the first cohort of migrant workers. These workers face policies of exclusion, occupational hazards, and economic disadvantage during their continuous migration, which accumulate over their life course and make them more likely to fall into poverty and accumulate higher health risks as they age.

**Figure 1:**
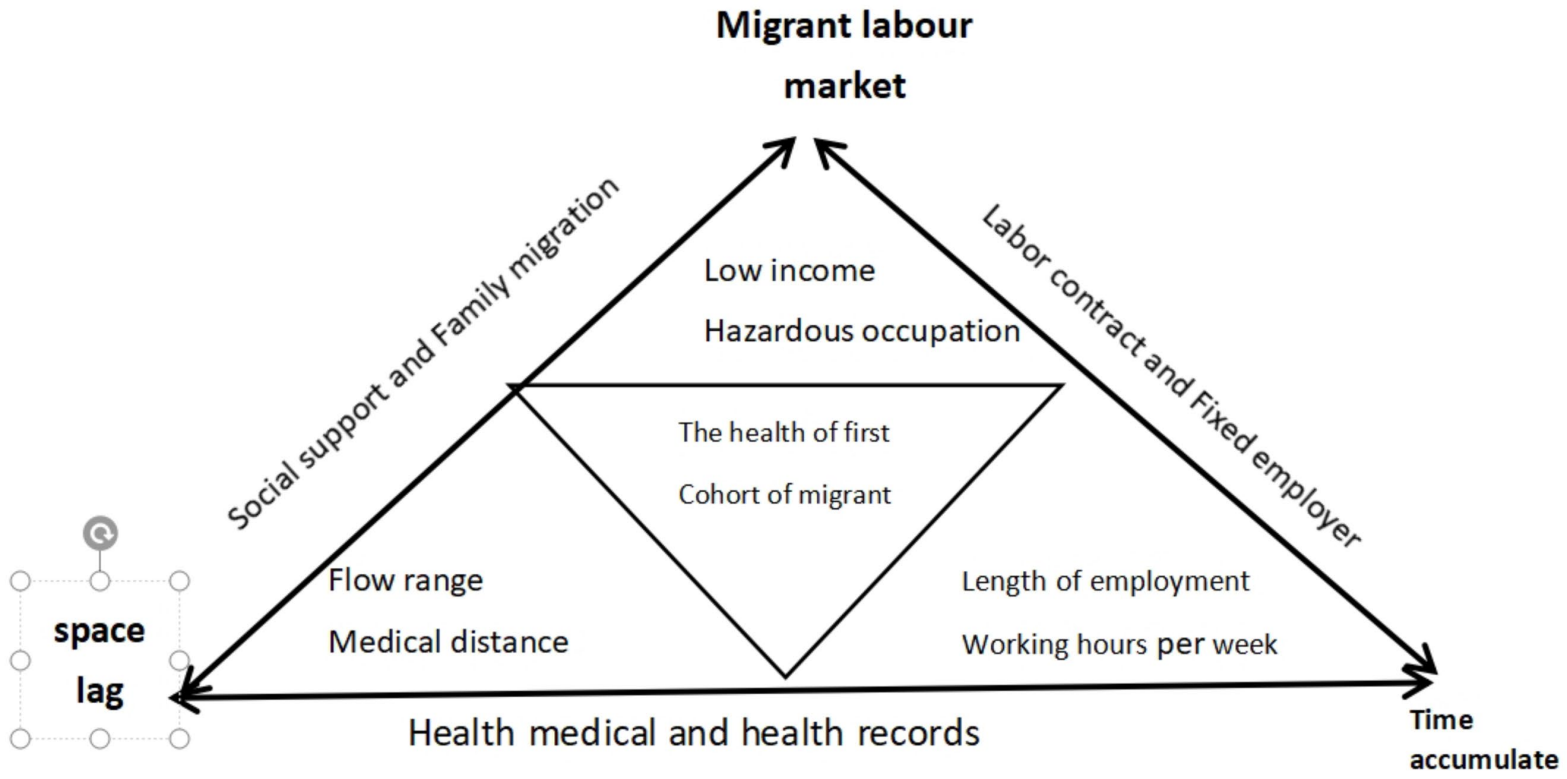
The conceptual model of migrant workers’ cumulative disadvantage in health.

From a specific analytical perspective, their work time was used as the longitudinal accumulation line, and their health care accessibility was used as the spatial lag zone to further clarify the factors that contribute to their health disadvantage accumulation process in both the longitudinal and horizontal boundaries. The process by which health disadvantages accumulate in the first cohort of migrant workers also involves constant interaction between individuals and their social environments, existing social policies, and economic incomes (see Figure 1).

### 2.2. Data sources and variables

The data for this study came from the China Migrants Dynamic Survey (2017), and we focused on rural migrant workers aged 40 and above. The specific definition used for inclusion in our study was those over 40 years old with a rural household registration. Jin Xiaoyi et al. defined the first cohort migrant workers in terms of their physical health, job type, and social security. They found that migrant workers over the age of 45 face significant pressures in employment, medical care, and income. In the working life of migrant workers, their income reaches its peak at the age of 35, after which it stabilizes and then declines (66). This decline happens earlier than for urban residents, who maintain their peak income until the age of 45, indicating that migrant workers lose job opportunities and earning capacity too early in the job market. The first cohort of migrant workers who are over 50 years of age faced amplified health risks and market livelihood risks, and their ability to resist risks is weakened as a result. Their opportunities to continue working in urban areas decreased, and their livelihood risks increased. For the purposes of this study, rural migrant workers over 40 years old were selected. After processing the variables, the resulting sample contained 36,668 observations. The basic characteristics of the sample were as follows: the average age of the respondents was 47.19 years; their average duration of education was 8.04 years; on average, they worked 60.10 h weekly; they had moved to an average of 2 cities; their logarithmic average income was CNY 8.024; 38% of the respondents were engaged in hazardous occupations; 46.1% had had an illness in the past year; 87.4% had insurance; 70.3% had received health education; 28.2% had created a health file; 32.6% had moved for economic reasons; 50.5% had moved together with their family; 35.8% reported having social exchanges locally; and 25.5% had signed labor contracts. Among them, male migrant workers accounted for 62.5% of all the respondents, and married migrant workers accounted for 94.5% of all the respondents.

### 2.3. Variable setting and measurement

#### 2.3.1. Dependent variable

In the research design of this study, the health status of migrant workers was considered the dependent variable, and self-assessment by the workers was used to obtain the data (67). Although there may have been some measurement error in self-reported health status, previous studies have suggested that it is a better indicator for predicting health status and is more robust than objective health measures (68). In the 2017 National Dynamic Monitoring Survey of the Health and Family Planning of the Floating Population questionnaire, the health status of migrant workers was divided into four categories: healthy, basically healthy, unhealthy but able to live independently, and unable to live independently. In this study, migrant workers who were classified as “basically healthy” were considered to have potential health risks or suboptimal health status (69), Disease is often regarded as a deviation from the normal standards of physical and mental well-being, based on common sense and everyday experience. Conversely, good health is defined as the absence of illness (70) and those classified as “unhealthy but able to live independently” and “unable to live independently” were combined into the category of “unhealthy status” and assigned a value of 0. Those classified as “healthy” were defined as having a “healthy status” and assigned a value of 1.

#### 2.3.2. Explanatory variables

We measured the explanatory variables considering three aspects.

Firstly, with respect to time indicators, this study examined the cumulative relationship between the health status of the first cohort of migrant workers and the duration of migration and weekly working hours. The duration of migrant workers’ migration and weekly working hours were used as indicators to measure the dimensions of their work time and weekly working hours. The time when they first left their registered household was used as an indicator to measure the duration of their migration. The duration of migration was calculated as 2017 minus the answer to question 302. The data from question 201 in the questionnaire were used as a variable to measure weekly working hours.^1^

Secondly, the relationship between health and medical service accessibility was examined considering the dimension of spatial differences in medical services for migrant workers. This study used the floating range of the first cohort of migrant workers and the distance to medical and nearby medical service points as variables. These two variables represented the accessibility of medical services for migrant workers.

Since the survey data were collected from our sample in 2017, considering that the medical insurance available to farmers was the new rural cooperative medical insurance or the urban resident medical insurance, this study did not use where migrant workers participated in medical insurance as a variable. Instead, the floating range of the first cohort of migrant workers was taken as a spatial variable to examine the distance from their home to their place of insurance, which was used as an indicator of the impact of health and medical service accessibility on the health of the first cohort of migrant workers. For this variable, out-of-province migration was assigned a value of 1, while non-provincial migration was assigned a value of 0.

The second spatial indicator reflects the impact of medical service accessibility on the health of rural migrant workers. In theory, the longer it takes for rural migrant workers to travel from their place of residence to the nearest medical service institution, the worse the accessibility of public services and basic health conditions are, and the lower the health maintenance level of rural migrant workers becomes, which affects their health. The distance from a respondent’s place of residence to their nearest medical service institution was coded as follows: within 15 min = 1; 15–30 min (inclusive) = 2; 30 min (exclusive)–1 h (inclusive) = 3; more than 1 h = 4.

Thirdly, the relationship between the accumulation of disadvantages in the first cohort of migrant workers and their health was examined considering the dimensions of their occupational characteristics and monthly income. The monthly income indicator was measured by the question, “How much was your wage income/pure income in the previous month (or previous employment)?” To avoid errors in the questionnaire and data, we performed a logarithmic transformation of the data. The respondents’ main occupations were measured by the question, “What is your current main occupation?.” We divided the occupations of the first cohort of migrant workers into hazardous and non-hazardous occupations based on the occupation’s degree of harm to the body. Where hazardous occupations = 1 and non-hazardous occupations = 0. Our occupational classification is primarily based on the theory of “skill segmentation” in the vertical labor market (71). In the labor market, employment situations can be categorized into non-routine cognitive, non-routine manual, routine cognitive, and routine manual occupations (72, 73). Building upon this framework and considering the Chinese labor market context, as well as the employment realities of migrant workers, this study further subdivides the employment of migrant workers into routine cognitive work and routine manual work. Following the design of previous relevant studies, occupations involving high pollution, high damage, high risk, and intense physical labor in industries such as processing, manufacturing, and construction are classified as hazardous work that poses significant health risks. Other occupations are classified as routine cognitive work, which is considered non-hazardous (72, 74).

#### 2.3.3. Control variables

To more accurately analyze the lagging and cumulative factors of the first cohort of migrant workers’ health in time and space, this study introduced three sets of control variables and gradually incorporated them to observe the factors affecting the health of the first cohort of migrant workers.

The first set of control variables includes the personal characteristics of the migrant workers, such as their age, years of education, marital status, and occupation, in order to reflect the individual factors that affect the health of the first cohort of migrant workers.

The second set of control variables reflects the family and social support factors of the first cohort of migrant workers, including mobility patterns, whether they moved with their families, and social communication, to reflect the influence of social support and family conditions on the health of the first cohort of migrant workers.

The third set of control variables reflects the personal medical accessibility of the first cohort of migrant workers, including whether they had established resident medical health records, whether they had received medical health education, etc., to reflect the relationship between medical accessibility for the first cohort of migrant workers and their health (Table 1).

**Table 1:** Statistical description of the variables (*N* = 36,668).

Variable	Assignment or definition	Observed value	Mean (ratio)	Standard deviation
Dependent variable				
Self-rated health	Healthy = 1, unhealthy = 0	36,668	0.756	0.429
Independent variables				
Working hours per week	Migrant workers work hours per week	36,665	60.104	18.990
Length of employment	The time when migrant workers leave their hometown	36,668	15.130	8.942
Mobility outside the province	Out-of-province = 1, non-provincial =0	36,668	0.592	0.492
Distance from medical institutions	Within 15 min = 1, for the 15–30 min = 2, for 30 min–1 h = 3, over 1 h = 4	36,668	1.190 (83.5, 14.9, 1.75, 0.26)	0.457
Hazardous occupation	Yes = 1, no = 0	36,668	0.380	0.485
Monthly income	Migrant worker wage income takes logarithmic treatment	36,060	8.024	0.608
Control variables				
Married	Yes = 1, no = 0	36,668	0.945	0.227
Gender	Male = 1, female = 0	36,668	0.625	0.484
Age	Actual age of migrant workers	36,668	47.185	5.621
Level of education	Illiteracy = 0, elementary school = 6, middle school = 9, senior middle school = 12; junior college = 15, undergraduate college = 16, graduate student = 20	36,668	8.040 (2.84, 14.30, 43.66, 21.9, 10.46, 6.42, 0.52)	2.830
The number of floatings	Number of cities where migrant workers have worked	36,665	2.134	2.530
Illness within a year	Yes = 1, no = 0	36,668	0.461	0.498
Health education	Yes = 1, no = 0	33,867	0.703	0.457
Health records	Yes = 1, no = 0	33,867	0.282	0.450
The floating of reason	Economic reasons = 1, social reasons = 0	36,668	0.326	0.469
Family migration	Yes = 1, no = 0	36,668	0.505	0.500
Sign a labor contract	Yes = 1, no = 0	36,668	0.255	0.436
Fixed employer	Yes = 1, no = 0	36,668	0.383	0.487

The percentages of each category are shown in parentheses for the multi-category variables. For the binary variables, the mean represents the percentage of the first category, while the proportion of the second category is omitted.

### 2.4. Data analysis methods

In order to test our hypothesis, a logit probability model was employed, with the health variable as a dummy variable, two levels as healthy and unhealthy. $Health_{i}$ values are 1 and 0, where 1 represents a healthy status and 0 represents an unhealthy status.

The probability of being in a healthy state:


$$P\left(health_{i}=1\right)=F\left(\alpha +\beta X_{i}\right)=\frac{1}{1+\exp\left[-\left(\alpha +\beta X_{i}\right)\right]},$$

and


$$\ln\frac{P\left(health_{i}=1\right)}{P\left(health_{i}=0\right)}=\alpha +\beta X_{i}.$$

The logit probability model was set up as follows:


(1) $$\log ithealth=i\ln\pi_{i}=\alpha +\beta X_{i}+\mu_{i},\pi_{i}=\frac{P\left(health_{i}=1\right)}{P\left(health_{i}=0\right)}$$

Here, $\pi_{i}$ is the odds ratio or probability ratio. $X_{i}$ represents the set formed by various explanatory variables, β represents the set formed by the coefficients of the various explanatory variables, and $u_{i}$ represents the random disturbance term.

The logistic regression model, compared to the tobit and probit models, is the most widely used model in current applications. The cumulative probability function of the logistic model is continuously differentiable, and it has better out-of-sample predictive performance. Due to its non-linear and explicit probability characteristics, the logistic model can be solved quickly. When there are no changes in the choice set of the model but only changes in the levels of variables, it is convenient to calculate the probabilities of being healthy or unhealthy in the new environment. The logistic model has the property of Independence of Irrelevant Alternatives (IIA), which assumes that the odds ratios of unrelated alternatives are independent. Therefore, when the health status of migrant workers can have multiple possibilities, the inclusion of additional health conditions does not affect the odds ratios of other health conditions. Thus, Model (1) can be easily extended to accommodate multiple health conditions. In light of this, in the robustness tests of Hypotheses 1 and 2 in Section 4, we will classify the health conditions as follows: $Health_{i}$ are set as follows: 1 – unable to perform daily activities, 2 – unhealthy but able to perform daily activities, 3 – basic health, and 4 – healthy.

In order to accurately estimate the parameter β , this study used a stepwise approach to add variables and observe changes in coefficients after each regression to assess the robustness of parameter estimates, drawing on Acemoglu et al. (75). Additionally, the study employed Baron and Kenny’s framework for analyzing moderation effects by gradually adding control variables and observing changes in the regression coefficients and their significance for both the main and moderator variables (interaction terms), to explore the reasons for the formation of the vulnerability of migrant workers (76). Similarly, based on a regression equation with the first cohort of migrant workers’ self-assessed health status as the dependent variable and variables with spatiotemporal characteristics, such as migration duration and medical insurance participation location, as independent variables, we constructed a regression equation by successively adding control variables, such as M1 and M2. We then compared the changes in coefficients between the latter and former equations, thereby analyzing the explanatory power of each set of variables, including differences in self-assessed health status among the first cohort of migrant workers. The OLS method used for obtaining the regression coefficients may have suffered from omitted variable bias, but since the omitted variables were mostly the same for the two regression equations compared (except for the addition of variable M in the latter equation), the impact of omitted variable bias would have been limited (77).

## 3. Analysis of data results

### 3.1. Descriptive statistics of health of first cohort of migrant workers

In this study, the definition of the first cohort of migrant workers referred to 36,668 individuals who were over 40 years old, had a rural household registration, and worked as employees. Among them, 31,344 individuals self-reported their physical health as good, accounting for 70.37% of the sample.^2^ This indicated that the physical health of the first cohort of migrant workers was relatively good, but their self-reported health was lower than that of the new generation of migrant workers. This is partly due to the aging of the first cohort leading to a higher degree of health loss. On the other hand, the logic behind the migration of the first cohort of migrant workers was different from that of second-generation migrant workers. The former aimed to obtain economic benefits, while the latter aimed to gain developmental opportunities. Therefore, their family responsibilities and ethical obligations were different. The first cohort of migrant workers adhered to the traditional culture of filial piety and had intergenerational family support and development responsibilities. Therefore, they rarely used their income for personal consumption, especially for health expenses, and delayed their health needs as much as possible in order to obtain maximum income, which led to greater health problems for themselves.

Furthermore, in terms of working hours, only 17.05% of the first cohort of migrant workers worked 40 h or less per week, which was lower than the average of 21.05% for all migrant workers in this period of time. The percentage of first-generation workers who worked between 40 and 60 h per week was 38.11%, which was also lower than the average of 40.91% for all migrant workers. The percentage of first-generation workers who worked more than 60 h per week was 44.74%, which was higher than the average of 38.04% for all migrant workers (77). From the statistical data, it can be seen that the average time that the first cohort of migrant workers spent working was lower than the average for migrant workers during standard working hours, or slightly higher working hours per week. However, their average working time was much higher than that of all migrant workers when they worked more than 60 h per week. Overall, 80% of migrant workers worked overtime, with almost no rest time. If calculated based on 1 day of rest on the weekend, their daily working time was more than 10 h. Excessive working hours seriously eroded the physical health of the first cohort of migrant workers.

### 3.2. Spatiotemporal extrusion regression analysis of the first cohort of migrant workers under the accumulation of disadvantages

The health issues of the first cohort of migrant workers included the long-term accumulation of disadvantages and the result of pressures on health caused by spatial and temporal lag from a policy perspective. The essential process by which these disadvantages accumulated lay in the economic and occupational vulnerabilities of these migrant workers – namely, the health disadvantages caused by engaging in heavy physical labor while working outside of their rural homes. Therefore, this accumulation process was not only a time-based accumulation; it was also affected by multidimensional factors. To ensure the robustness of the estimation results and to avoid the interference of multicollinearity, four models were produced by gradually introducing explanatory variables and control variables. All four models showed a significant negative correlation between the duration and range of migration and health. The estimation results are presented in Table 2.

**Table 2:** Regression analysis of the health and mobility of the first cohort of migrant workers.

	Model 1	Model 2	Model 3	Model 4
	Health	Health	Health	Health
	b/se	b/se	b/se	b/se
Health				
Hazardous occupation	−0.079^**^	−0.147^***^	−0.096^**^	−0.088^**^
	(0.026)	(0.028)	(0.030)	(0.030)
Floating outside the province	−0.108^***^	−0.119^***^	−0.130^***^	−0.124^***^
	(0.017)	(0.018)	(0.018)	(0.018)
Distance from medical institutions	−0.198^***^	−0.185^***^	−0.177^***^	−0.174^***^
	(0.026)	(0.028)	(0.028)	(0.028)
Length of employment	−0.018^***^	−0.019^***^	−0.018^***^	−0.018^***^
	(0.001)	(0.001)	(0.002)	(0.002)
Working hours per week	−0.002^**^	−0.002^**^	−0.003^***^	−0.003^***^
	(0.001)	(0.001)	(0.001)	(0.001)
Monthly income	0.516^***^	0.353^***^	0.343^***^	0.332^***^
	(0.021)	(0.022)	(0.022)	(0.022)
Income change	0.053^**^	0.093^***^	0.113^***^	0.090^***^
	(0.020)	(0.021)	(0.022)	(0.022)
Medical education		0.056	0.056	0.010
		(0.029)	(0.029)	(0.031)
Health records		0.111^***^	0.112^***^	0.043
		(0.031)	(0.031)	(0.033)
Gender		0.224^***^	0.228^***^	0.243^***^
		(0.029)	(0.029)	(0.029)
Age		−0.052^***^	−0.053^***^	−0.052^***^
		(0.002)	(0.002)	(0.002)
Level of education		0.029^***^	0.027^***^	0.021^***^
		(0.005)	(0.005)	(0.005)
Married		0.098	0.092	0.090
		(0.057)	(0.058)	(0.058)
Family migration			−0.245^***^	−0.244^***^
			(0.033)	(0.033)
The floating of reason			−0.068^*^	−0.271^***^
			(0.031)	(0.042)
The number of floating			0.017^**^	0.017^**^
			(0.006)	(0.006)
Fixed employers				0.166^***^
				(0.045)
Sign labor contract				0.207^***^
				(0.041)
_cons	−2.236^***^	0.982^***^	1.143^***^	1.213^***^
	(0.177)	(0.240)	(0.242)	(0.242)
*N*	36,057	33,307	33,307	33,307
*r*3				
*p*	2.08e-190	6.14e-303	1.29e-310	8.40e-323

*t* statistics in parentheses. *p < 0.05, **p < 0.01, ***p < 0.001.

Model 1 is a basic regression model that used external working time, engagement in hazardous occupations, out-of-province mobility, weekly working hours and distance from the hospital as core explanatory variables to verify Hypotheses 1–3.^3^ The regression results showed that the weekly working hours and external working time (duration away from home) were significantly negatively correlated with health status at the 1% and 0.1% levels, respectively. This indicated that, the longer the weekly working hours and the duration away from home, the lower the probability of good health. The regression results supported Hypothesis 1. Out-of-province mobility and distance from the hospital were both significantly negative at the 1% level, supporting Hypothesis 2. Engagement in hazardous occupations was significantly negative at the 1% level, also supporting Hypothesis 2. Monthly income was significantly positive at the 1% level, indicating that, the higher the income, the greater the probability of good health. This suggested that individuals with higher incomes had stronger health maintenance abilities, helping to alleviate the accumulation of health disadvantages. The regression results of Model 1 fully supported the hypothesis of the health disadvantage accumulation of migrant workers. Although scholars have conducted related research on the health status and working time of migrant workers, there is a lack of empirical research with large-sample survey data. Our sample, taken from the first cohort of migrant workers in China, generally had an external working time of more than 20 years, and they engaged in more 4D-type work, with overtime as a common phenomenon. This study further clarified the process by which the health disadvantages of the first cohort of migrant workers accumulated by selecting specific research objects from nationwide data.

Considering the working life history of the first cohort of migrant workers, they were a typical “low-skilled, low-educated, low-income, and high-risk” population. They are known as “second-class citizens” in the job market (78). Due to the special history of the earliest group of migrant workers, the first cohort of migrant workers experienced a “process of recognition” when entering the city, which means they gradually moved from being excluded to being recognized (79). In the low-income and high-loss labor environments, the longer the first cohort of migrant workers’ external mobility time, the greater their health losses, and the lower their health self-evaluation (9). From this perspective, the external mobility time of migrant workers is essentially a process of health disadvantage accumulation. That is, their investment in health maintenance was minimal, while their health losses increased day by day. Under such an accumulation of health disadvantages, the health outcomes of the first cohort of migrant workers were obviously not optimal.

Model 2 added medical education, health records, gender, age, marital status, and other first-type control variables of our first designated category to control for the individual characteristics.^4^ The regression results showed that the signs and significance of the core variables in Model 1 did not differ significantly from those in Model 2, and the conclusions of Hypotheses 1–2 remained robust. In Model 2, the estimates of the mobility range (out-of-province mobility) and mobility time (working time) variables were negative and significant at the 0.1% level. We can therefore conclude that, the longer the external mobility time of migrant workers, the worse their health self-evaluation. That is, the larger the mobility ranges and the longer the durations of time away from home, the greater the time and economic costs of medical expense reimbursement, thus exacerbating the accumulation of health disadvantages for migrant workers.

To further control the influence of the selection characteristics of the first cohort of migrant workers, the second- and third-category control variables, such as family migration, reasons for migration, the number of migrations, and the signing of labor contracts, were added to Models 3 and 4. After these control variables were added, there were no significant changes in the parameter symbols and significance levels of the core explanatory variables in the models, and the estimation results were robust. The mobility range and mobility time variables remained significantly negative at the 1% level.

According to the various models in Table 2, the parameter symbols of the income variables and income change variables were significantly positive at the 5% level, indicating that income plays a crucial role in health maintenance. However, the income of the first cohort of migrant workers was generally lower than that of urban residents, and the role of income in health maintenance was not strong enough to offset the cumulative damage to health caused by the weak position of migrant workers. Data showed that medical services only became a necessity for rural residents as late as 2004. This transformation was critical in the establishment of the new rural cooperative medical insurance, which allowed rural residents to enjoy corresponding medical service benefits even on extremely low incomes. The income level of migrant workers peaked at the age of 35 and then showed a stable decline, falling earlier than urban residents’ income, indicating that migrant workers lose employment opportunities and income capacity too early in the job market. Their lower income, coupled with their low health maintenance capacity, further exacerbates the accumulated health disadvantages of the first cohort of migrant workers. At the same time, in terms of the age dimension, there was also a significant negative correlation between age and health within the first cohort of migrant workers, showing that health declines with increasing age. Of course, even if occupational and economic factors are excluded, a negative correlation between age and health problems regarding individual physiological functions also exists. However, for the first cohort of migrant workers, due to their unique life course, their occupations and low economic status undoubtedly exacerbate the health disadvantages caused by the correlation between age and health beyond what is due to natural factors.

According to Models 3 and 4, their reason for migration is significantly related to health, which also verified that the critical factors for the first cohort of migrant workers’ mobility were economic rather than social. Migrant workers only choose areas and industries with higher incomes, and do not consider other impact of other factors on health.^5^ This is the rational strategy of “survival first” for migrant workers, and it is almost their only choice, indicating that migrant workers may sacrifice health for income. In terms of the number of migrations, health status was significantly positively correlated with the number of migrations these workers made, indicating that, the more they migrated, the higher their self-rated health, which partially validated the “salmon bias” explanation; that is, the healthier the migrant workers are, the more they will chase better opportunities by constantly moving. Because they have better physical health, they are more willing to invest time and other costs in searching for jobs. Those who are unhealthy can only lower their expectations, go with the floating, reduce the number of migrations they make, and pursue short-term benefits, thus becoming unable to change their weak position and likely to fall into a predicament as they age and their physical condition declines, increasing the possibility of a vicious cycle.

According to Models 3 and 4, the health level of migrant workers was significantly negatively associated with family migration at the 0.1% level. The self-rated health of the first cohort of migrant workers undertaking migrations with their the families was significantly lower than that of non-family migration migrant workers who did not migrate with their families. Whether family migration affects health is the result of comprehensive family dynamic decision making, which is related not only to health but also to income. Insufficient income cannot meet the expenses of a family, including those for children’s education.

Due to the collinearity between the family migration mode and income, adding the family migration variable increases the variance of parameter estimation and reduces the precision of our estimate of the effect of the income variable due to collinearity. The standard error relative to the parameter’s estimated value thus increases in this situation.^6^

The results obtained regarding family migration also raised some questions for the existing research in the academic community. In the existing research, rural-to-urban migration is seen as an important way to improve the health and mental health of migrant workers (80, 81). However, the results of this study showed that, for the first cohort of migrant workers, migrating with their family had a negative impact on their health. The family stress theory may better explain the impact of family migration on the health of the first cohort of migrant workers. The first cohort of migrant workers were in the low-end job market with low incomes, and they tried to maximize the comparative benefits between rural and urban areas by squeezing their own consumption. However, regarding home-based migration, a family’s living expenses in the city are obviously higher than those in the countryside. Single migrant workers can be more frugal in terms of accommodation, food, and daily life. However, when they migrate with their family, their offspring need to study close to where the parents work, incurring more expenses. Therefore, the first cohort of migrant workers had to further compress their health expenditures, seek more dangerous or worse jobs to strive for higher incomes, and provide more economic support for their offspring’s development in the city. Family stress, therefore, further strengthened the health vulnerability of the first cohort of migrant workers.

Model 4 added labor contracts and fixed employers as control variables to on the basis of the previous model to further examine the health status and job stability of the first cohort of migrant workers. The regression results were significant, such that having a formal contract and a fixed employer could significantly improve health at the 0.1% level. In general, having a formal labor contract may increase income. A formal contract and a fixed employer also mean a safe working environment and good social relationships, which can increase confidence and a sense of security, and improve health. Migrant workers with higher incomes can live in more convenient environments, including locations that are closer to medical institutions. The presence of a labor contract and a fixed employer has a key impact on the health of migrant workers. The benefit of labor contracts and fixed employers for migrant workers lies in whether they can provide relatively stable health security and future job expectations for migrant workers. Existing studies also show that the health status of migrant workers is positively correlated with the signing of labor contracts. In signed labor contracts, the medical insurance of migrant workers can be reimbursed locally and can be included in the employee’s medical insurance, thereby solving the problems of the spatial lag and non-portability of medical insurance. However, the low actual contract signing rate among migrant workers may hinder the realization of these benefits.

## 4. Tests of the robustness of the accumulation of health disadvantages and spatio temporal lag in the first cohort of migrant workers

The regression model results above demonstrate that there are significant spatial differences in the health status of the first cohort of migrant workers, which can be observed with the gradual addition of other factors. In essence, these differences relate to the current medical insurance system, suggesting that the New Rural Cooperative Medical Scheme (NRCMS) exhibits a spatial lag effect in maintaining the health of migrant workers. Additionally, the health of the first cohort of migrant workers is subject to the cumulative effect of time vulnerability. More loss-intensive and longer jobs result in greater health losses for migrant workers and poorer self-evaluations of health. To further test the robustness of the baseline regression results, this study changed the setting of the dependent variable and conducted a robustness test by replacing the dependent variable and re-regressing it. The results of the robustness test shown in Table 3 indicated that changing the setting of the dependent variable did not affect the baseline regression results. Specifically, even when controlling for other factors, the health of the first cohort of migrant workers is still affected by the spatiotemporal lag represented by the typical variables of migration range and duration, and there is also a time vulnerability accumulation mechanism in terms of health. The robustness test supported Hypotheses 1, 2.

**Table 3:** Tests of the robustness of the accumulation of health disadvantages and spatio temporal lag in the first cohort of migrant workers.

	Model 5	Model 6	Model 7
	Health 1	Health 2	Health 3
	b/se	b/se	b/se
Main			
Floating outside the province	−0.071^***^	−0.101^***^	−0.130^***^
	(0.016)	(0.015)	(0.018)
Distance from medical institutions	0.033	−0.068^**^	−0.193^***^
	(0.025)	(0.024)	(0.028)
Length of employment	−0.019^***^	−0.022^***^	−0.018^***^
	(0.001)	(0.001)	(0.002)
Working hours per week	−0.007^***^	−0.005^***^	−0.002^**^
	(0.001)	(0.001)	(0.001)
Monthly income.	0.167^***^	0.286^***^	0.362^***^
	(0.020)	(0.019)	(0.023)
Income change	0.046^*^	0.072^***^	0.102^***^
	(0.018)	(0.017)	(0.022)
Hazardous occupation	−0.152^***^	−0.134^***^	−0.090^**^
	(0.025)	(0.023)	(0.030)
Medical education	−0.068^**^	−0.029	−0.056
	(0.025)	(0.024)	(0.029)
Health records	0.137^***^	0.124^***^	0.107^***^
	(0.026)	(0.024)	(0.031)
Gender	0.148^***^	0.194^***^	0.231^***^
	(0.025)	(0.023)	(0.029)
Age	−0.002	−0.027^***^	−0.052^***^
	(0.002)	(0.002)	(0.002)
Level of education	0.008	0.021^***^	0.028^***^
	(0.004)	(0.004)	(0.005)
Married	−0.264^***^	−0.124^*^	−0.096
	(0.051)	(0.048)	(0.058)
Family migration	−0.369^***^	−0.350^***^	−0.246^***^
	(0.029)	(0.026)	(0.033)
The floating of reason	−0.251^***^	−0.242^***^	−0.179^***^
	(0.030)	(0.029)	(0.035)
The number of floating	0.017^**^	0.001	0.018^**^
	(0.006)	(0.005)	(0.006)
Sign labor contract	0.076^*^	0.059^*^	0.269^***^
	(0.032)	(0.029)	(0.037)
cut1	−0.018	−0.490^*^	0.959^***^
	(0.215)	(0.206)	(0.245)
cut2		1.186^***^	3.248^***^
		(0.206)	(0.248)
cut3		2.840^***^	8.965^***^
		(0.208)	(0.560)
cut4		4.681^***^	
		(0.217)	
*N*	33,307	33,307	33,307
*r*3			
*p*	5.30e-200	4.94e-324	0

*t* statistics in parentheses. *p < 0.05, **p < 0.01, ***p < 0.001. Models 5 and 6 were estimated using the ordinal selection model.

Model 5 re-categorized the health status of migrant workers and redefined the health variables as follows: 1 = unable to take care of oneself, 2 = unhealthy but able to take care of oneself, 3 = basically healthy, and 4 = healthy. To distinguish the health variables in Model 5 from those in Table 2, the health variable in Model 5 was named Health 1. The results showed that the insurance location, migration duration, social interactions, and income from family migration are significantly related to health status. The parameter symbols did not change from Model 2.

Model 6 experienced a slight increase in the standard error, resulting in the t-value just falling below the critical threshold of 5%. In Model 7, an illness was used as an indicator of health, with 1 = no illness and 0 = having an illness. The health variable was named Health3. After adding various control variables and regressing the dependent variable of Health3, the results showed that mobility duration, mobility range, income, and other factors were significantly correlated with having an illness, and medical education and medical records significantly influenced the illness status of migrant workers. Consistent with many models in Table 2, income status also affected the health maintenance ability of the first cohort of migrant workers, with lower income being associated with poorer health status.

## 5. Further discussion of income and health

Tables 2, 3 show that the variables regarding the reasons for migration are significantly negative, revealing that migrant workers who move for economic and income reasons may harm their health. However, this does not necessarily mean that increasing income will harm health; instead, it indicates that the relationship between income and health is conditional. Of course, most people in the labor market meet this condition, leading to its neglect in the health domain, but the first cohort of migrant workers is an exception.

Income, as a key variable, plays a significant role in the health evaluation of migrant workers in the context of their accumulated health vulnerabilities. In academic research on the relationship between migrant workers’ health and income, most studies have indicated that lower income has a greater impact on health (63, 66). However, there are also some workers who sacrifice their health to obtain a higher income by extending working hours or engaging in dangerous 4D work (37, 39). The bidirectional relationship between health and income has always been a focus of discussion in research on the relationship between the two factors.

In this study, to test Hypothesis 3, to further reduce the bidirectional influence between working hours, income, and health, two methods were adopted. One was the analysis of moderating effects, using income as a moderating variable to analyze whether income has a detrimental effect on health when working hours are extended. The other method was to find proxy variables for sacrificing health to obtain income. Income is obtained by labor under the condition of unchanged health status. Labor input is represented by working hours, and income obtained by labor input is income obtained by sacrificing health. The residual obtained by regressing income on variables such as working hours represented the income obtained by sacrificing health. This residual method has been widely used in related studies (82–84). The specific equation is as follows:


(2) $$\ln income_{i}=\beta_{1}+\beta_{2}worktiome_{i}+\sum \delta_{j}z_{j}+u_{i}$$

Other variables influencing income are included in Eq. 2. The estimated residuals from Eq. 1 can serve as a proxy for the portion of income obtained at the cost of sacrificing health. Subsequently, using the residuals as the explanatory variable for health, we analyzed the factors contributing to the vulnerability of the health of the first cohort of migrant workers from the perspectives of policy-induced spatial lag and the duration of migrant workers’ out floating. The regression results are shown in Table 4.

**Table 4:** Testing the sacrifice of health for economic income among the first cohort of migrant workers.

	Model 8	Model 9	Model 10	Model 11	Model 12
Variables	Lincome	Health	Health	Health	Health
Monthly income		0.123***	0.666***	0.601***	0.681***
		(4.953)	(15.72)	(15.17)	(16.07)
Sacrifice the income earned from your health			−0.968***		−0.616***
			(−15.06)		(−5.620)
Interaction term between sacrificing health and weekly work hours				−0.0135***	−0.00601***
				(−14.88)	(−3.841)
Working hours per week	0.00156***	−0.00178**			
	(7.889)	(−2.076)			
Floating outside the province	0.116***	0.171***	0.0632*	0.0736**	0.0595*
	(17.34)	(5.386)	(1.907)	(2.235)	(1.797)
New rural cooperative location	0.0413***	0.151***	0.129***	0.123**	0.124**
	(4.067)	(3.178)	(2.655)	(2.521)	(2.549)
Length of employment		−0.216***	−0.240***	−0.234***	−0.239***
		(−6.139)	(−6.668)	(−6.519)	(−6.647)
Distance from medical institutions	0.0205***	−0.155***	−0.176***	−0.171***	−0.175***
	(2.757)	(−4.639)	(−5.142)	(−4.988)	(−5.108)
Married	0.0516***	0.0228	−0.115	−0.105	−0.121
	(3.562)	(0.304)	(−1.492)	(−1.368)	(−1.573)
Gender	0.245***	0.205***	0.0203	0.0300	0.0105
	(35.32)	(5.942)	(0.549)	(0.821)	(0.285)
Age	−0.0905***	−0.349***	−0.274***	−0.283***	−0.272***
	(−21.71)	(−17.60)	(−13.04)	(−13.60)	(−12.95)
Level of education	0.0282***	0.0230***	0.00286	0.00410	0.00165
	(21.15)	(4.017)	(0.471)	(0.682)	(0.272)
Ill within a year	−0.0362***	−0.843***	−0.825***	−0.826***	−0.824***
	(−5.514)	(−26.57)	(−25.53)	(−25.55)	(−25.48)
Participate in the new rural insurance	−0.144***	−0.218***	−0.126**	−0.133**	−0.121**
	(−14.31)	(−3.838)	(−2.193)	(−2.319)	(−2.115)
Health education		0.0778**	0.103***	0.0989***	0.103***
		(2.231)	(2.900)	(2.778)	(2.885)
Health records	−0.0254***	0.0971***	0.119***	0.111***	0.117***
	(−3.574)	(2.706)	(3.251)	(3.050)	(3.202)
The floating of reason	0.0359***	0.128***	0.0233	0.00975	0.0109
	(4.565)	(3.517)	(0.661)	(0.276)	(0.308)
Family migration	0.0628***	−0.0181	−0.0656*	−0.0674*	−0.0700**
	(9.282)	(−0.534)	(−1.893)	(−1.947)	(−2.019)
Family economic status		0.284***	0.508***	0.475***	0.511***
		(8.427)	(13.15)	(12.60)	(13.22)
Family difficulties		0.608	0.469	0.510	0.476
		(1.074)	(0.804)	(0.872)	(0.813)
Local social interaction	0.0614***	0.0677**	0.0176	0.0215	0.0149
	(9.141)	(2.054)	(0.521)	(0.637)	(0.442)
Engaged in the industry		−0.0284			
		(−0.658)			
The health of migrant workers	0.119***				
	(15.23)				
Whether there is contracted land	−0.0318***				
	(−4.609)				
Are there any dividends	0.105***				
	(4.862)				
Constant	7.555***	−1.020	−3.523***	−3.163***	−3.553***
	(271.3)	(−1.612)	(−5.240)	(−4.740)	(−5.270)
Observations	31,299	22,472	22,029	22,029	22,029
*R*-squared	0.14	0.0644	0.0024	0.7646	0.1172

*t* statistics in parentheses. *p < 0.05, **p < 0.01, ***p < 0.001.

According to Table 4, the income equation of Model 9 included a series of variables that influence income, such as working hours, mobility range, occupational characteristics, human capital (education, health), social capital (whether workers experience social interactions in the local area), production capital (whether there is insurance coverage and whether there are dividends), and social security level (whether workers are enrolled in social security). Therefore, other factors influencing income are hidden in the residuals, including income obtained at the cost of sacrificing health. Thus, the residuals obtained from Model 9 can be used as a proxy for income obtained at the cost of sacrificing health. In order to accurately characterize the vulnerable position of the first cohort of migrant workers who sacrificed their health for income out of necessity, Model 9 excluded variables related to hazardous industries or occupations (hazardous occupations) that may be detrimental to health and included these detrimental factors in the residuals. Model 10 did not include the variable of income obtained at the cost of sacrificing health, while Model 11 included this variable. Model 12 included the interaction term between income obtained at the cost of sacrificing health and weekly working hours, to reflect the dual effect of engaging in hazardous occupations such as 4D works and damaging health by increasing working hours. Model 13 included both the variable of income obtained at the cost of sacrificing health and the interaction term between this variable and weekly working hours.

The estimation results of Models 10–13 showed that income obtained at the cost of sacrificing health was significantly negative at the 1% significance level after controlling for wage income and other variables, verifying the fact that migrant workers generally exchange health for income, and confirming the accumulation of vulnerabilities among migrant workers. Hypothesis 3 has been validated. The interaction term between income obtained at the cost of sacrificing health and weekly working hours in Models 12 and 13 was also significantly negative at the 0.1% level, indicating that the working environment of migrant workers (e.g., 4D work) is correlated with weekly working hours, and the combination of the two further reduces health levels, or that the detrimental working environment worsens the health levels of migrant workers by prolonging working hours. The estimation results of the interaction term also suggested that migrant workers do not receive sufficient income compensation for their poor working environment. If the compensation were high enough, migrant workers would choose to reduce working hours to restore their health and maintain labor force levels. However, in reality, the detrimental working environment and long working hours have a cumulative effect, and migrant workers do not choose to substitute working environment for working hours, but rather continue to increase working hours and choose poorer working environments to ensure necessary income. Furthermore, for these individuals, it also shows that income does not significantly increase with working hours, working environment, or work intensity. In terms of the employment sector, the first cohort of migrant workers were mainly concentrated in labor-intensive, low-income industries, engaging in high-intensity, high-risk, low-wage jobs. In some construction sites, about 90% of the workers were the first cohort of migrant workers. These characteristics of the first cohort of migrant workers make them typical representatives of excessive labor and low income in the job market (85).

Tables 2, 3 respectively validate the hypotheses presented earlier through regression models and robustness tests, respectively. Specifically, we found that the health of the first cohort of migrant workers is subject to spatial lag and vulnerable accumulation mechanisms. The spatial lag was reflected in a significant negative correlation between the range of mobility and self-rated health of the first cohort of migrant workers. Vulnerability accumulation was primarily shaped by factors such as low income, hours of overtime worked per week, 4D-type jobs, and time spent away from home, which cumulatively contributed to the health vulnerability of the first cohort of migrant workers. Furthermore, this vulnerability became increasingly apparent as this cohort aged. Throughout their working lives, the market disadvantage and excessive labor involved in their jobs contributed to the process of the accumulation of health vulnerabilities.

## 6. Discussion and conclusion

### 6.1. Discussion

Our study is the first to utilize nationwide large-scale data to demonstrate the phenomenon of accumulated vulnerability in the health status of first-cohort migrant workers. Previous studies have primarily focused on the social relationships and economic income of migrant workers (2, 3), without considering the unique health issues faced by first cohort of migrant workers，the issue of accumulating health vulnerabilities. For migrant workers, the accumulation of vulnerabilities poses a harm to their health that is greater than the sum of its parts (1 + 1 > 2). Previous research has only considered the issue of individual vulnerabilities (4, 86), while the combination of various disadvantages can lead them to actively choose work environments that are detrimental to their health in order to earn income. This short-term behavior and the lack of consideration for their future well-being result in a pessimistic outlook for their later years in life. Additionally, our study discusses the relationship between the familial migration of migrant workers and their health, we found that when migrant workers migrate with their families, it has an impact on their health. Our study expand the research perspective on family-based migration of migrant workers. Internationally, scholars have often discussed the health issues of cross-border migration and have also shown concern for the health and occupational issues of immigrants (9, 58). However, they have not conducted data-driven comparative analyses on the relationship between immigrants’ occupations and health Furthermore, there has been limited discussion on the portability of policies related to Chinese immigrant health issues and the influence of traditional Chinese culture, such as filial piety culture, on their health factors. Specifically, first-cohort migrant workers primarily allocate their income to sustaining their family’s income, often allocating fewer funds for their own health issues.

### 6.2. Policy implications

From a policy perspective, the tension between the high mobility of migrant workers and the regional limitations of the medical insurance system creates a rigid situation in which the use of medical insurance by migrant workers is limited by their mobility. This rigidity significantly inhibits the health maintenance ability of migrant workers, resulting in the inclusion of spatial lag in the typical characteristics of health maintenance for migrant workers. Additionally, the limitations imposed by the New Cooperative Medical Scheme (NCMS) on the place of residence and household registration further exacerbate the dilemma between the mobility of rural residents and their access to medical resources, leading to significant difficulties for migrant workers in obtaining medical care. In reality, many individuals in the first cohort of migrant workers faced a difficult decision between maintaining their health and sustaining their livelihood. For them, giving up their mobility was equivalent to giving up their only means of accessing economic resources. Thus, many migrant workers chose to sacrifice their medical benefits to ensure the sustainability of their livelihood (87).

In the context of China’s aging society, it is crucial to further stimulate the labor capacity of middle-aged and older adult groups, and break the non-benign cycle of health, mobility, and employment among the first cohort of migrant workers. It is necessary to reduce the impact of health problems on China’s labor supply market, and enhance the medical security system and health maintenance ability of the first cohort of migrant workers. Therefore, the following policy recommendations are proposed:

First, at the level of the overall planning of urban and rural residents’ social medical insurance, efforts should be made to achieve provincial-level or large regional-level coordination. Anhui Province’s migrant worker floating is mainly concentrated in the Yangtze River Delta region, where the cross-regional coordination of residents’ social medical insurance can be achieved. The Bohai Economic Rim could also address the mobility and medical needs of migrant workers in the north, making medical insurance more adaptable to the high-mobility characteristics of migrant workers, and eliminating the binary tension between their native residence home and their need for high mobility. This will provide convenience in the health maintenance of migrant workers and eliminate the effect of lag in the treatment of health issues.

Second, a special medical initiative action for migrant workers should be launched to enhance the health maintenance ability of the first cohort of migrant workers. Research has shown that establishing health records and receiving relevant health education in the workplace could significantly enhance the health maintenance ability of the first cohort of migrant workers. Establishing nearby community medical locations could also significantly improve their health status. It is necessary to enforce a health examination system for the first cohort of migrant workers, with their workplaces as the first responsible party. Special projects for the health maintenance of migrant workers should be established, and enterprises and social forces should be mobilized to participate.

Third, health knowledge and routine medical care for the first cohort of migrant workers should be promoted to enhance their medical literacy. Improving the health literacy of migrant workers can help achieve the early diagnosis and treatment of health problems and eliminate the accumulation of health vulnerabilities. Following the concept of promoting health education and health literacy for the “whole population” and “full life cycle,” targeted screening for health risks and activities to improve health risk awareness should be conducted for older migrant workers.

### 6.3. Study limitations and contributions

#### 6.3.1. Study limitations

This study was based China Migrants Dynamic Survey (2017). This study has some limitations.

First, this is a cross-sectional study, which to some extent has limited our chance to explore the comparison of health status among migrant workers before and after migration due to the lack of respondents health information before 2017.

Meanwhile, interpretation of the findings implying causality should take caution. A longitudinal study may yield a better understanding of the relationship between migrant workers’ mobility and their health.

Second, the existing data mainly focuses on the health of migrant workers, and lacks the health data of urban populations during the same period, which limits our opportunity to make comparisons between the health status of migrant workers and urban populations.

Third, there may be other factors that affect the health of the first cohort of migrant workers, but our study is limited to the questionnaire items, which also affects our study of the specificity of the health status of the first cohort of migrant workers.

#### 6.3.2. Contributions

Despite of its limitations, this study contributes to the existing literature by providing a more comprehensive understanding of the factors that contribute to the poor health outcomes of Chinese migrant workers. Specifically, the study highlights the unequal access to social rights, particularly healthcare, as a major underlying cause of migrant workers’ poor health beyond the limitations of China’s universal healthcare system. By shedding light on this issue, the study underscores the need for systemic changes to address the structural barriers that hinder healthcare access for migrant workers and promote greater equity in healthcare delivery in China.

## Data availability statement

The original contributions presented in the study are included in the article/supplementary material, further inquiries can be directed to the corresponding author.

## Author contributions

FQ and QK made the conceptualization, designed methodology, wrote the main manuscript text and reviewed and edited. FQ, QK, and DF prepared Tables 1–4. FQ was responsible for funding acquisition. All authors contributed to the article and approved the submitted version.

## Funding

This research was funded by Social Science Fund of Anhui Province, grant number: AHSKY2022D172.

## Conflict of interest

The authors declare that the research was conducted in the absence of any commercial or financial relationships that could be construed as a potential conflict of interest.

## Publisher’s note

All claims expressed in this article are solely those of the authors and do not necessarily represent those of their affiliated organizations, or those of the publisher, the editors and the reviewers. Any product that may be evaluated in this article, or claim that may be made by its manufacturer, is not guaranteed or endorsed by the publisher.

## Footnotes

^1^When was the first time you left your domicile (county level)? Have you ever done more than 1 h of paid work in the week before the May Day holiday this year?

^2^Data source: Data of 2017 China Migrants Dynamic Survey.

^3^Due to the primary goal of income for the first-generation migrant workers in their migration, income is a critical control variable related to the core explanatory variable. Omitting this variable would result in significant estimation bias. Therefore, Model 1 includes income as a control variable.

^4^These control variables not only influence the health level of migrant workers but also have correlations with the core explanatory variables. For instance, in cases where families do not migrate, migrant workers face challenges in taking care of their children when they relocate to distant provinces, and in such situations, left-behind family members are responsible for farming the land. Being in close proximity to home during busy farming seasons allows for immediate support. Furthermore, migrant workers who have established health records and received health education have a better understanding of their health issues and are aware of the occupational requirements and hazards. They can choose to avoid certain positions or reduce their time spent in such occupations. For instance, individuals with respiratory conditions tend to avoid jobs with high levels of dust.

^5^Due to the correlation between household migration patterns and income, adding household migration variables can increase the variance of parameter estimates due to collinearity, thus reducing the accuracy of parameter estimates. The inclusion of household migration variables also decreases the estimated value of income variables affected by collinearity and increases the relative standard error. As a result, the actual contract signing rate may be even lower.

^6^The actual contract signing rate may be lower.

## References

[1] Cai Z Wang D. Sustainability and labor contribution of China's economic growth. Econ Res. (1999) 10:62–8.

[2] Wang XZ. From 'survival' to 'recognition': the problem of migrant workers from the perspective of citizenship rights. Soc Stud. (2009) 1:121–38.

[3] Chen YF. Migrant workers: institutional arrangements and identity recognition. Soc Stud. (2005) 3:119–32.

[4] Zhou XG Lu M. Health of migrants: China's achievement or regret? Econ Res J. (2016) 3:79–98.

[5] National Bureau of Statistics. "Statistical communique of the People's Republic of China on the 2021 National Economic and Social Development." (2022) Available at: http://www.stats.gov.cn/tjsj/zxfb/202202/t20220227_1827960.html

[6] National Bureau of Statistics. Monitoring Survey Report on Migrant Workers. (2022) Available at: http://www.gov.cn/xinwen/2022-04/29/content_5688043.htm

[7] Wang CG. The relationship between social identity of New generation rural floating population and urban-rural integration. Soc Stud. (2001) 3:63–76.

[8] Song LF. The formation, trend and countermeasures of the "migrant workers wave". Chinese Soc Sci. (1995) 4:78–91.

[9] Liu CJ Cheng JL. The intergenerational differences and citizenization of migrant workers in China. Econ Netw. (2007) 4:18–21. doi: 10.3969/j.issn.1007-7685.2007.04.006

[10] Xu CX. Ways of migrating into the city and 0ccupational mobility of migrant workers: comparative analysis of two generations of migrant workers. Youth Stud. (2010) 3:1–12.

[11] Lee K McGuinness C Kawakami T. Research on occupational safety and health for migrant workers in five Asia and the Pacific countries: Australia, Republic of Korea, Malaysia, Singapore and Thailand. Geneva: International Labour Organization (2011).

[12] Qiu FX. Analysis of the accessibility of health services for the first generation of migrant workers from the perspective of building a healthy China. J Anhui Normal Univ (Soc Sci Ed). (2023) 2:81–92. doi: 10.14182/j.cnki.j.anu.2023.02.009

[13] Jacquelyn Flaskerud H Winslow BJ. Conceptualizing vulnerable populations health-related research. Nurs Res. (1998) 47:69–78. doi: 10.1097/00006199-199803000-00005, PMID: 9536190

[14] Dale D. Cumulative advantage/disadvantage and the life course: cross-fertilizing age and social science theory. J Gerontol. (2003) 6:S327–37. doi: 10.1093/geronb/58.6.S327 14614120

[15] Bauman LJ Silver EJ Stein REK. Cumulative social disadvantage and child health. Pediatrics. (2006) 117:1321–8. doi: 10.1542/peds.2005-1647, PMID: 16585330

[16] O'Rand AM. The precious and the precocious: understanding cumulative disadvantage and cumulative advantage over the life course. Gerontologist. (1996) 36:230–8. doi: 10.1093/geront/36.2.230, PMID: 8920094

[17] Falci CD Mortimer JT Noel HJ. Parental timing and depressive symptoms in early adulthood. Adv Life Course Res. (2010) 15:1–10. doi: 10.1016/j.alcr.2010.05.001, PMID: 21197392 PMC3011890

[18] Merton RK. Social theory and social structure. New York: Free Press, a division of Macmillan Pub (1967).

[19] Leight SB. The application of a vulnerable populations conceptual model to rural health. Public Health Nurs. (2003) 20:440–8. doi: 10.1046/j.1525-1446.2003.20604.x, PMID: 14629675

[20] England P Garcia-Beaulieu C Ross M. Women’s employment among blacks, whites, and three groups of Latinas: do more privileged women have higher employment? Gend Soc. (2004) 18:494–509. doi: 10.1177/0891243204265632

[21] Guralnik JM Butterworth S Wadsworth MEJ Kuh D. Childhood socioeconomic status predicts physical functioning a half century later. J Gerontol Ser A Biol Med Sci. (2006) 61:694–701. doi: 10.1093/gerona/61.7.694, PMID: 16870631

[22] Hamil-Luker J O’Rand AM. Gender differences in the link between childhood socioeconomic conditions and heart attack risk in adulthood. Demography. (2007) 44:137–58. doi: 10.1353/dem.2007.0004, PMID: 17461340

[23] O'Rand AM Jenifer HL. Processes of cumulative adversity: childhood disadvantage and increased risk of heart attack across the life course. J Gerontol Ser B Psychol Sci Soc Sci. (2005) 2:S117–24. doi: 10.1093/geronb/60.Special_Issue_2.S117 16251582

[24] Galobardes B John WL Smith GD. Childhood socioeconomic circumstances and cause-specific mortality in adulthood: systematic review and interpretation. Epidemiol Rev. (2004) 26:7–21. doi: 10.1093/epirev/mxh008, PMID: 15234944

[25] Riley MW Foner A Waring J. Aging and society: a sociology of age stratification. Manhattan, NY: Russell Sage Foundation (1972).

[26] Clausen J. The continuing problem of defining mental deficiency. J Spec Educ. (1972) 6:97–106. doi: 10.1177/002246697200600113, PMID: 32491480

[27] Li Q Deng JW. Social change and personal development: paradigms and methods of life course research. Soc Stud. (1999) 6:1–18.

[28] Dannefer D. Cumulative advantage /disadvantage and the life course: cross-fertilizing age and social science theory. J Gerontol Ser B Psychol Sci Soc Sci. (2003) 58:S327–37. doi: 10.1093/geronb/58.6.S327, PMID: 14614120

[29] Guo YH Chang AS. Life course and social security: a sociological exploration of the life course of laid-off and unemployed workers. Chinese Soc Sci. (2005) 5:93–107.

[30] Bao LP. Analysis of time perception in life course theory. Soc Stud. (2005) 4:120–33. doi: 10.19934/j.cnki.shxyj.2005.04.005

[31] Jiao KS Bao ZM. Social change, life course, and elderly health. Soc Stud. (2020) 35:149–69. doi: 10.19934/j.cnki.shxyj.2005.04.005

[32] Elder J Glen H Avshalom C. Human development and social change: an emerging perspective on the life course In: Bolger N Caspi A Downey G Moorehouse M, editors. Persons in context: *developmental processes*. New York: Cambridge University Press (1988). 77–113.

[33] Elman C Angela MO. The effects of social origins, life events, and institutional sorting on adults’ school transitions. J Soc Sci Res. (2007) 36:1276–99. doi: 10.1016/j.ssresearch.2006.11.001

[34] Glasgow N Brown DL. Older, rural and poo In: Coward RT Krout JA, editors. Aging in rural settings: life circumstances and distinctive features. New York: Springer Publishing (1998). 187–205.

[35] Lee LF. Health and wage: a simultaneous equation model with multiple discrete indicators. Int Econ Rev. (1982):199–221.

[36] Stern S. Semiparametric estimates of the supply and demands effects of the disability on labor force participation. J Econ. (1996) 71:49–70. doi: 10.1016/0304-4076(94)01694-1

[37] Liu GE William H Dow Fu AZ Akin JS. Health human capital and income growth in China. China Econ Q. (2004) 4:101–18.

[38] Strauss J Thomas D. Human resources: empirical modeling of household and family decisions In: Behrman JR Srinivasan TN, editors. Handbook of development economics, vol. 3. Amsterdam: North-Holland (1995). 1883–2023. doi: 10.1016/S1573-4471(05)80006-3

[39] Wei Z. The role of health on off-farm employment and wage decision. Econ Res J. (2004) 2:64–74.

[40] Zhang CC. Empirical analysis on impact of health change on labor supply and income. Econ Rev. (2011) 4:79–88.

[41] Zhou F. Income and health of urban migrant workers: effects of occupational status. Econ Forum. (2009) 22:49–52. doi: 10.3969/j.issn.1003-3580.2009.22.016

[42] Yuan HN. Health and income of migrant workers in cities – evidence from a survey of migrant workers in Beijing. J Manag World. (2009) 5:56–66.

[43] Qin LJ Chen B Qin XZ. Analysis of the impact of health on the income of migrant workers going out to work. World Econ Papers. (2013) 6:110–20.

[44] Qian L Chen J. Rely on education or health: the influence of the two kinds of human capitals on migrant workers' income and its differentiation effect. Xinjiang State Farms Econ. (2018) 2:11–9.

[45] Ren H Ren QQ. Demand intensity and participation behavior of migrant workers' medical insurance: an explanation for the deviation phenomenon. Study Pract. (2020) 3:61–75.

[46] Fu DY Li NX Gao B. Factors influencing migrant workers' health insurance selection in the process of urbanization: based on the date of Chengdu. J Sichuan Univ (Philos Soc Sci Edn). (2018) 3:63–8. doi: 10.3969/j.issn.1006-0766.2018.03.010

[47] Feng J Yu YY. Income inequality and health in rural China. Econ Res J. (2007) 1:79–88.

[48] Christopher B Stern N. Productivity, wages and nutrition, part I, the theory. J Dev Econ. (1978) 5:331–62. doi: 10.1016/0304-3878(78)90016-0

[49] Grossman M. On the concept of health capital and the demand for health. J Polit Econ. (1972) 80:223–55. doi: 10.1086/259880, PMID: 37415702

[50] Lu WC Li YL. Theoretical reasoning on health rights of migrant workers: a perspective of environmental justice. Chinese J Popul Sci. (2009) 3:13–20.

[51] Xiong GL Wu J Wang F Cheng MJ. Pay attention to the health risks of migrant workers. Med Soc. (2006) 2:1–5.

[52] Huang QB Sa ZH. The association between working hours and physical and mental health among rural – urban migrant workers in China. Chin J Health Psychol. (2015) 3:358–62. doi: 10.13342/j.cnki.cjhp.2015.03.012

[53] Zhou XG Lu M. The rural migrant workers' health: China's success or regret? China J Econ. (2016) 3:79–98.

[54] Liu Y. The dynamic change of Chinese migrant workers’ health – evidence from CHNS (1997-2006) survey data. J Xinjiang Univ (Philos Soc Sci). (2011) 6:33–37. doi: 10.3969/j.issn.1000-2820.2011.06.007

[55] Mi SH Li BZ Zhu QB. The impact of social capital on the health status of rural – urban migrant workers. Issues Agric Econ. (2016) 9:42–53.

[56] Feng J. Health needs and medical security system construction: a study of rural China, Shanghai. Shanghai People’s Publishing House. (2009) 79–176.

[57] Ye MH. Is the medical service to rural residents luxury or necessity? Based on rural and urban comparison of medical demand income elasticity:1990—2009. Issues Agric. (2011) 6:30–5.

[58] Li XJ Wang ZH Lin ZP. The impact of new rural cooperative medical care on farmers' medical treatment behavior and health – based on the analysis of different income levels. World Econ Papers. (2012) 3:58–75.

[59] Wang J Zheng J Wang P Qi L. Migration and health in China: bridging the gaps in policy aims and the reality of medical services to migrants. J Public Adm. (2014) 4:29–45.

[60] McDonald JT Kennedy S. Insights into the healthy immigrant effect: health status and health service use of immigrants to Canada. Soc Sci Med. (2004) 59:1613–27. doi: 10.1016/j.socscimed.2004.02.004, PMID: 15279920

[61] Marmot MG Adelstein AM Bulusu L. Lessons from the study of immigrant mortality. Lancet. (1984) 323:1455–7. doi: 10.1016/S0140-6736(84)91943-3 6145889

[62] Arifin E.N. Ananta A. Punpuing S. Impact of migration on health in Kanchanaburi, Thailand. *International Population Conferenc*e 2005, 18–23.

[63] Liu JJ. An empirical analysis of the impact of health risks on rural labor migration. J Hulunbuir Univ. (2013) 5:10–4. doi: 10.3969/j.issn.1009-4601.2013.05.005

[64] Yin QS Wang W Wang P. Circular effect analysis of income and health status of rural residents in China: An empirical analysis based on CHNS data. Economist. (2011) 11:43–51.

[65] Crystal S Shea D. Cumulative advantage, cumulative disadvantage, and inequality among elderly people. Gerontologist. (1990) 30:437–43. doi: 10.1093/geront/30.4.437, PMID: 2394380

[66] Jin XY Hu ZY Gu DD. Who are the elderly migrant workers: data analysis based on the monitoring survey of floating population. Manag Rev. (2018) 7:271–80.

[67] Alpert PT. Self-perception of social isolation and loneliness in older adults. Home Health Care Manag Pract. (2017) 29:249–52. doi: 10.1177/1084822317728265, PMID: 37232847

[68] Poortinga W. Social relations or social capital? Individual and community health effects of bonding social capita. Soc Sci Med. (2006) 1:255–70. doi: 10.1016/j.socscimed.2005.11.039 16427171

[69] Yi YY Song XW. Study on the importance of factors affecting the health of floating population in China: empirical analysis based on random forest model. Northwest Popul J. (2020) 4:15–26. doi: 10.15884/j.cnki.issn.1007-0672.2020.04.002

[70] Calnan M. The health locus of control: an empirical test. Health Promot Int. (1987) 4:323–30. doi: 10.1093/heapro/2.4.323

[71] Ehrenberg RG Smith RS. Modern labor economics, theory and public policy. Boston: Addison-Wesley Educational Publishers Inc. (1997).

[72] Cortes GM Jaimovich N Siu HE. Disappearing routine jobs: who, how, and why. J Monet Econ. (2017) 91:69–87. doi: 10.1016/j.jmoneco.2017.09.006

[73] Cain GG. The challenge of segmented labor market theories to orthodox theory: a survey. J Econ Lit. (1976) 14:1215–1257.

[74] Zhang S Shi X. Market segmentation, capital deepening, and education deepening: further thoughts on employment issues. J Yunnan Univ (Soc Sci). (2003) 5:70–76. doi: 10.3969/j.issn.1671-7511.2003.05.010

[75] Acemoglu D Johnson S Robinson J Thaicharoen Y. Institutional causes, macroeconomic symptoms: volatility, crises and growth. J Monet Econ. (2003) 50:49–123. doi: 10.3386/w9124

[76] Baron R.M. Kenny D. A. The moderator-mediator variable distinction in social psychological research: conceptual, strategic, and statistical considerations. London: Chapman and Hall (1986), 51, 173–1182. 10.1037//0022-3514.51.6.1173 3806354

[77] Qi MZ Wang Y. Research on the socio-economic stratification of floating population in China. Chinese J Popul Sci. (2021) 6:110–123+128.

[78] Démurger S Gurgnd M Li A Yue X. Migrants as second-class workers in urban China? A decomposition analysis. J Comp Econ. (2009) 37:610–28. doi: 10.1016/j.jce.2009.04.008

[79] Wang XZ. From "survival" to "recognition": the problem of migrant workers in the field of citizenship. Sociol Res. (2009) 1:21–138.

[80] Liang H. The mental health status of migrant workers from the perspective of intergenerational differences. Popul Res. (2014) 4:87–100.

[81] Yu L Zhu Y. The impact of residential segregation on the health of migrant population: an analysis based on the 2014 dynamic monitoring data of migrants. Shandong Soc Sci. (2018) 6:120–8.

[82] Richardson S. Over-investment of free cash flow. Rev Acc Stud. (2020) 11:159–89.

[83] Hribar P Melessa SJ Small RC. Does managerial sentiment affects accrual estimates? Evidence from the banking industry. J Account Econ. (2017) 63:26–50. doi: 10.1016/j.jacceco.2016.10.001

[84] Xin BH Dou YZ Tao J. Confucian culture affects the stability of commercial banks. J Anhui Normal Univ (Hun Soc Sci). (2023) 1:129–143. doi: 10.14182/j.cnki.j.anu.2023.01.013

[85] Guo F.M. Zhang S.W. (2021). Does signing labor contracts help alleviate migrant workers? *World Economic* Conference 2021, 6, 1-16.

[86] Li DP Lu HY Wen XL. Labor time, social interaction, and the physical and mental health of migrant workers: an empirical analysis based on CGSS2013 data. Survey World. (2018) 3:40–5. doi: 10.13778/j.cnki.11-3705/c.2018.03.007

[87] Qiu FX Liu J Zhan HJ. Migration and health—freedom of movement and social benefits for Chinese migrant workers. Sustainability. (2021) 13:1–26. doi: 10.3390/su132212371

